# Emergent airway management outside of the operating room – a retrospective review of patient characteristics, complications and ICU stay

**DOI:** 10.1186/s12871-019-0894-4

**Published:** 2019-12-03

**Authors:** Uzung Yoon, Jeffrey Mojica, Matthew Wiltshire, Kara Segna, Michael Block, Anthony Pantoja, Marc Torjman, Elizabeth Wolo

**Affiliations:** 1 Department of Anesthesiology, Thomas Jefferson University Hospital, Suite 8290 Gibbon, 111 South 11th Street, Philadelphia, PA 19107 USA; 2 Department of Anesthesiology, Johns Hopkins University Hospital, Baltimore, MD USA

**Keywords:** Emergent airway, Outside the operating room, Intubation, Mortality, Cardiac arrest

## Abstract

**Background:**

Emergent airway management outside of the operating room is a high-risk procedure. Limited data exists about the indication and physiologic state of the patient at the time of intubation, the location in which it occurs, or patient outcomes afterward.

**Methods:**

We retrospectively collected data on all emergent airway management interventions performed outside of the operating room over a 6-month period. Documentation included intubation performance, and intubation related complications and mortality. Additional information including demographics, ASA-classification, comorbidities, hospital-stay, ICU-stay, and 30-day in-hospital mortality was obtained.

**Results:**

336 intubations were performed in 275 patients during the six-month period. The majority of intubations (*n* = 196, 58%) occurred in an ICU setting, and the rest 140 (42%) occurred on a normal floor or in a remote location. The mean admission ASA status was 3.6 ± 0.5, age 60 ± 16 years, and BMI 30 ± 9 kg/m^2^. Chest X-rays performed immediately after intubation showed main stem intubation in 3.3% (*n* = 9). Two immediate (within 20 min after intubation) intubation related cardiac arrest/mortality events were identified. The 30-day in-hospital mortality was 31.6% (*n* = 87), the overall in-hospital mortality was 37.1% (*n* = 102), the mean hospital stay was 22 ± 20 days, and the mean ICU-stay was 14 days (13.9 ± 0.9, CI 12.1–15.8) with a 7.3% ICU-readmission rate.

**Conclusion:**

Patients requiring emergent airway management are a high-risk patient population with multiple comorbidities and high ASA scores on admission. Only a small number of intubation-related complications were reported but ICU length of stay was high.

## Background

Emergent airway management is required outside of the operating room (OR) in every hospital setting. It is an inherently higher risk procedure when compared to controlled OR settings [1]. In the OR, most intubations are done under an elective, controlled environment and under supervision of attending anaesthesiologists. Intubations outside of the OR are performed under less ideal conditions which can lack appropriate personnel, equipment and monitoring devices. Outside OR intubations are performed in the ICU, general floor, emergency room or remote locations. Very little is known about the number of intubations performed and subsequent outcome of those patients.

Patients requiring emergent intubation are frequently hemodynamically unstable, hypoxic, and rarely NPO. History, physical exam, and information handoff by the primary care team is often incomplete or limited in an emergent airway setting. There is also limited time to perform an adequate airway exam.

Emergent intubation complications often result from compromised patient’s physiologic status, limited reserve, limited airway evaluation, difficult airway management, and inability to pre-oxygenate the patient. A 3% mortality rate within 30 min of intubation has been reported in the intensive care unit (ICU) setting [2]. Several studies have documented an 8–12% incidence of difficult intubation in the emergent setting [3–5] compared to an incidence of 5.8% during elective intubation in the OR [6].

Limited data exist about outside OR intubations including patient comorbidity on admission and physiologic state at the time of intubation and shortly thereafter. Also little is known about the length of ICU-stay and in-hospital mortality of those patient population.

The objective of this study was to evaluate the patient characteristics, intubation performance and outcome after emergent airway management occurring outside of the OR.

## Methods

Following institutional review board approval and waived consent, data for all airway intubations were collected retrospectively over a 6-month period. At our institution, the anaesthesiology department is responsible for all airway management outside of the OR except in the emergency department. This includes the acute care floors (587 beds), medical-ICU (23 beds), surgical-ICU (17 beds), cardiac-ICU (17 beds), neurosurgery-ICU (14 beds), and remote locations (CT, MRI, cardiac-catheterization-laboratory, interventional-radiology, endoscopy).

The airway response resident responded to the emergent airway when there is a page received to an emergency pager. This included code blue, rapid response (RRT), Anaesthesia STAT, level 1 trauma, or elective intubation request which were defined as:

Code blue was announced for cardiopulmonary arrest or other life-threatening events.

RRT was announced for non-life threatening but significant change in physiologic status and/or vital signs that requires urgent intervention by the RRT team. Anesthesia STAT was announced for urgent intubation in a hemodynamically stabile patient. (e.g. self extubation, GI bleeding). Elective intubation was announced in patients with stabile vital signs requiring non-urgent intubation (e.g. elective procedure outside of the OR, anticipation of potential respiratory failure, airway protection).

Level 1 trauma was announced for injury with signs of shock or respiratory distress, penetrating injury to head, neck, torso, fascial or neck injury with actual or potential airway compromise or traumatic cardiac arrest.

For intubation an anaesthesia attending and/or any training level resident was available for assistance in airway management. The induction medication kit was centralized by pharmacy and brought by the nursing staff to the bedside. Induction kit medications contained etomidate, rocuronium, succinylcholine, phenylephrine, and ephedrine. Sugammadex was not available at this time as part of the standard induction medication kit.

Intubation was confirmed by 6 breath trial capnometer color change and bilateral breath sounds. After intubation, documentation was completed by the anaesthesia resident performing or supervising the intubation. Defined data points were time of intubation, location, indication for intubation, number of attempts, laryngoscopic view, ETCO_2_ detection, medication use, vital signs, and complications. Additionally, we retrospectively performed a complete search of the electronic health and imaging records for every intubated patient.

Immediate intubation-related mortality was defined as the event that occurred during or within 30 min of intubation without clear indication of other causes. Extubation was defined as either endotracheal extubation or tracheostomy placement. The primary outcome measure of the study was immediate intubation related complication and mortality (< 30 min). Secondary outcome measures were ICU stay, ICU readmission rate, hospital stay, 30-day in-hospital mortality. Additionally, demographics including age, sex, BMI, ASA status and comorbidity were collected on initial admission. No recalculation was performed for patients who had reintubation events. Cerebral performance category was upon cischarge was calculated to measure the extent and severity of neurological impairment and disability (1. Full recovery, 2. Moderate cerebral disability but independent in activities of daily living 3. Severe cerebral disability, dependent in activities of daily living, 4. Persistent vegetative state, 5. Brain dead).

Arithmetic mean, standard deviations, and 95% confidence intervals was used to report the patient’s demographics. Data were also reported as medians with interquartile range (IQR) when indicated. Statistical analyses were performed using Chi-Square, Fisher, and independent 2 tailed t-tests. Systat (Systat Software Inc., San Jose, CA) version 13 software was used.

## Results

### Demographics and clinical details

**Fig. 1:**
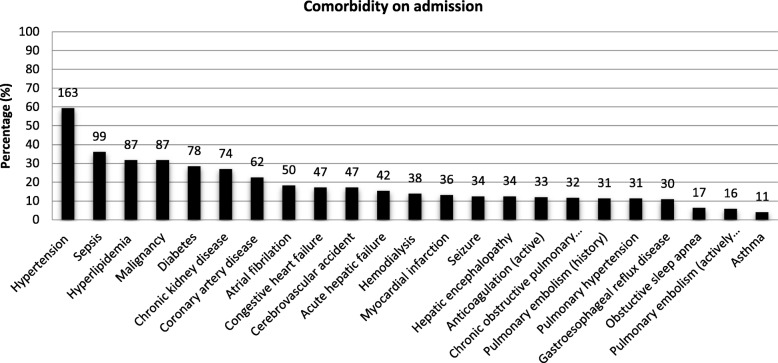
Comorbidity on admission in 275 patients (%)

Data for 352 emergent intubations were collected and reviewed. Due to lack of documentation, 16 patients were excluded. The final analysis included 336 intubations in 275 patients during the 6-month period. Reintubation occurred in 51 patients (18.5%). Overall 58% of the patients were male aged 59 ± 15 years with a mean admission ASA status of 3.6 ± 0.5 and BMI if 30 ± 9 kg/m^2^ (Table 1). The most common comorbidity was hypertension, followed by sepsis, hyperlipidaemia, and malignancy (Fig. 1). Airway management was requested for the following reasons: code blue (*n* = 28; 8.3%), rapid response team (*n* = 66; 19%), anaesthesia STAT (*n* = 106; 31.5%), and urgent intubation (*n* = 137; 40.8%). More than half of the intubations occurred in an ICU setting (*n* = 196; 58%), and the rest (*n* = 140; 42%) occurred on a normal floor or in a remote location.

**Table 1:** Characteristics of patients requiring emergent intubation outside the OR. (*n* = 275)

Demographics	
Age (years)	59.4 ± 15.4
Male	159 (57.8%)
Female	116 (42.2%)
Ethnicity	
White	179 (65.1%)
Black	65 (4.4%)
Hispanic	12 (1.5%)
Unknown	11 (23.6%)
Asian	4 (4%)
Height	169.7 ± 12.3 cm
Weight	86 ± 28.1 kg
BMI (overall)	30 ± 8.8 (kg/m^2^)
< 18.5 (underweight)	12 (4.4%)
18.5–24.9 (normal)	73 (26.5%)
25–29.9 (overweight)	85 (30.9%)
30–34.9 (moderate obese)	50 (18.2%)
35–39.9 (severely obese)	23 (8.4%)
≥ 40 (very severely obese)	32 (11.6%)
ASA classification on admission	3.6 ± 0.5
ASA 1	1 (0.3%)
ASA 2	4 (1.5%)
ASA 3	94 (34%)
ASA 4	176 (64%)
ASA 5	0 (0%)
Comorbidity on admission	
Hypertension	163 (48.5%)
Sepsis	99 (29.5%)
Hyperlipidemia	87 (25.9%)
Malignancy	87 (25.9%)
Diabetes	78 (23.2%)
Chronic kidney disease	74 (22%)
Coronary artery disease	62 (18.5%)
Atrial fibrillation	50 (14.9%)
Congestive heart failure	47 (14%)
Cerebrovascular accident	47 (14%)
Acute hepatic failure	42 (12.5%)
Hemodialysis	38 (11.3%)
Myocardial infarction	36 (10.7%)
Seizure	34 (10.1%)
Hepatic encephalophaty	34 (10.1%)
Anticoagulation (active)	33 (9.8%)
Chronic obstructive lung disease	32 (9.5%)
Pulmonary embolism (history)	31 (9.2%)
Pulmonary hypertension	31 (9.2%)
Gastroesophageal reflux disease	30 (8.9%)
Obstructive sleep apnea	17 (5.1%)
Pulmonary embolism (actively)	16 (4.8%)
Asthma	11 (3.3%)

### Indication for intubation

The most common indication for intubation was acute respiratory failure in 254 (75.6%) patients, followed by the need for intubation to perform an urgent or elective procedure outside of the OR in 36 (10.7%), airway protection in 24 (7.1%), self extubation in 19 (5.7%), and endotracheal tube exchange in 3 (0.9%). Intubation performance included location, time of event, oxygenation upon arrival, induction, medication used, ventilation, intubation device, grade, attempt, difficulty, and placed ETT size (Table 2).

**Table 2:** Intubation performance (n = 336)

	Number of patients (N = 336)	Percentage (%)
Indication for Intubation		
Acute respiratory failure	254	(75.6%)
Need for intubation to perform an urgent or elective procedure outside of the OR	36	(10.7%)
Airway protection	24	(7.1%)
Self extubation	19	(5.7%)
Endotracheal tube exchange	3	(0.9%)
Location		
ICU	196	(58%)
Non ICU (ward, remote location, trauma room)	140	(42%)
Timing of events		
6:00 AM - 6:00 PM	193	(57.4%)
6:00 PM - 6:00 AM	139	(41.4%)
Attending Present	13	(3.9%)
Oxygenation (upon arrival to scene)		
Non rebreather face mask	118	(35.1%)
Nasal cannula	87	(25.9%)
Bag mask ventilation	50	(14.9%)
BIPAP (Bilevel Positive Airway Pressure)	42	(12.5%)
Room air	14	(4.2%)
CPAP (Continuous Positive Airway Pressure)	3	(0.9%)
Face tent	1	(0.3%)
Patient was already Intubated	1	(0.3%)
Not documented	20	(6%)
Induction		
Standard intravenous induction	131	(39.0%)
RSI (rapid sequence induction)	176	(52.4%)
Ventilation (after induction)		
Easy ventilation	162	(48.2%)
Easy with airway adjunct	55	(16.4%)
Moderate difficult with airway adjunct	10	(3.0%)
Difficult	4	(1.2%)
Two person ventilation	24	(7.1%)
Unable to ventilate	2	(0.6%)
Not indicated	91	(27.1%)
Cricoid Pressure applied	170	(50.6%)
Cricoid Pressure not applied	159	(47.3%)
Medication		
Etomidate	281	(83.6%)
Propofol	24	(7.1%)
Ketamine	1	(0.3%)
No sedation medication for induction	31	(9.2%)
Rocuronium	277	(82.4%)
Succinylcholine	28	(8.3%)
No muscle relaxant for induction	28	(8.3%)
Phenylephrine	40	(11.9%)
Ephedrine	4	(1.2%)
Other	5	(1.5%)
Intubation device		
Mac blade	236	(70.2%)
MAC 3	86	(36.4%)
MAC 4	144	(61.0%)
Not reported	6	(2.5%)
Miller	0	(0%)
Video laryngoscope	92	(27.4%)
Glidescope® blade 3	63	(68.5%)
Glidescope® blade 4	25	(27.2%)
Not reported	4	(4.3%)
Laryngeal Mask Airway (LMA)	1	(0.3%)
Awake fiberoptic	5	(1.5%)
Surgical Airway	2	(0.6%)
Bougie	2	(0.6%)
Intubation Grade (Cormack-Lehane Grading)		
Grade 1. Full view of glottis	252	(75.0%)
Grade 2. Partial view of glottis	56	(16.7%)
Grade 3. Only epiglottis seen, none of glottis seen	20	(6.0%)
Grade 4. Neither glottis nor epiglottis seen	5	(1.5%)
Intubation attempt		
Attempts 1	296	(88.1%)
Attempts 2	31	(9.2%)
Attempts 3	7	(2.1%)
Attempts > 3	0	(0%)
Difficulty (Intubation Difficulty Scale)		
Easy	290	(86.3%)
Mod difficult	35	(10.4%)
Difficult	6	(1.8%)
Impossible	1	(0.3%)
Attempt aborted	0	(0%)
Intubation achieved	333	(99.1%)
ETT size (mm)		
5	1	(0.3%)
5.5	0	(0%)
6	1	(0.3%)
6.5	4	(1.2%)
7	48	(14.3%)
7.5	184	(54.8)
8	88	(26.2)
8.5	1	(0.3%)
Unknown	9	(2.7%)

### Post induction hemodynamics and intubation related complications

After induction, there was an average decrease of 2 mmHg (2.3 ± 1.6, CI − 5.3-0.8) in systolic blood pressure and an average increase in heart rate of 5 bpm (4.9 ± 1, CI 2.9–6.9) (Table 3). Chest X-rays performed immediately after intubation showed main stem intubations in 3.6% (*n* = 10). No dental injuries or unrecognized oesophageal intubations were identified. One new onset of a small apical pneumothorax was reported in one patient, with spontaneous resolution within 24 h. Intubation was atraumatic for most patients (*n* = 325; 96.7%). Intubation-related complications were reported in 5 (1.5%) of the intubated patients, and these complications consisted of: lip laceration (*n* = 2; 0.6%), tongue injury (*n* = 1; 0.3%), vomiting during induction (n = 1; 0.3%), and other (n = 1; 0.3%).

**Table 3:** Hemodynamic changes pre- and post-induction/ intubation

	(n = 336)			
	Pre intubation	Post intubation		
Systolic blood pressure (SBP)	130 ± 1.8	128 ± 1.8	Decreased 2.3 ± 1.6 mmHg, (CI −5.3-0.8)	*P* = 0.079
Diastolic blood pressure (DBP)	74 ± 0.9	74 ± 1	Decreased 0.4 ± 1.1 mmHg, (CI −2.5-1.7)	*P* = 0.411
Heart rate (HR)	105 ± 1	110 ± 1	Increased 4.9 ± 1 BPM, (CI 2.9–6.9)	*P* < 0.001

### Immediate complication and mortality after intubation

Two immediate complications events occurred wihtin 30 min of intubation. The first patient experienced ventricular fibrillation arrest 4 min after intubation with a CPR time of 45 min until expiration. The patient had a history of cardiomyopathy, EF 45%, severe pulmonary hypertension, COPD, coronary artery disease and was admitted for CHF exacerbation.

The second patient had pulseless electrical activity 17 min after intubation with a CPR time of 25 min until expiration. The patient had a history of non-ischemic cardiomyopathy status post multiple cardioversion, cryo-ablation and ICD placement, atrial fibrillation, aortic value replacement (for bicuspid aortic valve and aortic insufficiency), transient ischemic attack, and pericarditis. This patient was admitted with worsening heart failure, EF 15% complicated by stroke and ventricular tachycardia during their hospital stay.

### Intubation related morbidity and in-hospital mortality

**Fig. 2:**
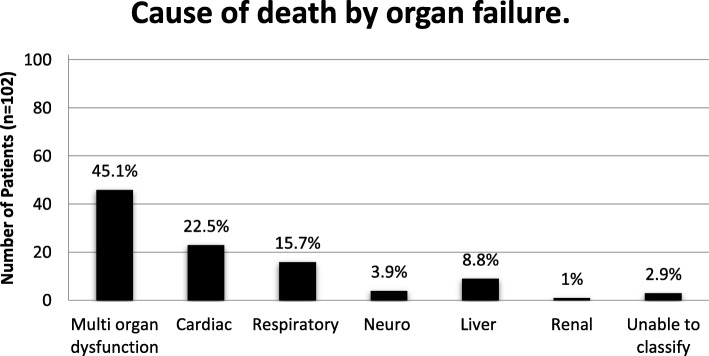
Cause of death by organ failure

33 (12%) patients had newly diagnosed pneumonia after intubation, and 64 patients (23.3%) required a tracheostomy placement after an average of 9.2 ± 7.4 days of intubation. The 30-day in-hospital mortality was 31.6% (*n* = 87), the overall in-hospital mortality was 37.1% (*n* = 102), the mean hospital stay was 22 ± 20 days, and the mean ICU-stay was 14 days (13.9 ± 0.9, CI 12.1–15.8) with a 7.3% ICU-readmission rate (Table 4). The most common reason for death was multi-organ dysfunction followed by cardiac and respiratory reasons (Fig. 2).

**Table 4:** Long-term outcome of patients after outside OR airway management

Complications and outcome	(n = 275)
Pneumonia	33 (12%)
Average intubation days	7.1 ± 8.8
Tracheostomy	64 (23.3%)
Average time until tracheostomy	9.2 ± 7.4
Hospital stay	22.3 ± 19.6 days
ICU stay	13.7 ± 15.3 days
ICU readmission rate	7.3%
Reintubations	112 out of 336 intubations (33.3%)
Reintubated patients	51 out of 275 patients (18.5%)
Mortality	
Overall mortality	102 (37.1%)
30-day in hospital mortality	87 (31.6%)
Cerebral performance category upon discharge	3.1 ± 1.6

Cerebral performance category:

1.Full recovery

2.Moderate cerebral disability but independent in activities of daily living

3.Severe cerebral disability, dependent in activities of daily living

4.Persistent vegetative state

5.Brain dead

## Discussion

### Intubation performance and difficult intubation

In this study, we found 88.1% of the intubations were accomplished on the first attempt. Stauffer et al. reported difficult airway management in 30% of intubations and Willich et al. in 20% [7, 8]. Martin et al. reported difficult airway management in 10% in of patients managed outside of the OR [9]. Most likely the lower incidence in this study is explained by the extensive airway training and simulation program we perform to prepare physcians for emergent airway managements outside the OR. The importance of airway education for airway management outside th eopreating room has been described by Rochlen et al. [10] In general, repeated attempts at tracheal intubation should be avoided because they increase the incidence of airway obstruction, leading to serious airway complications [11, 12].

### Intubation related complications

The immediate intubation-related outcome was low. Traumatic intubation was reported in only less than 1%. Our study showed bronchial intubation rate of 3.6%. The literature reports an ETT misplacement rate ranging from 4 to 28% [13–15]. Several studies have suggested inaccuracy of auscultation of bilateral breath sounds in determining proper ETT position. Anatomical variations such as large breasts, obesity, or barrel chests may make the assessment of auscultation and chest expansion more difficult. Additionally, with partial blockage of the mainstem bronchus breath sounds may be normal. To minimize the risk of bronchial intubation the top of the cuff should be seen to have just passed through the cords, the length of the tube noted at the lips and then secured. Cuff palpation at the sternal notch has been shown to effectively confirm ETT location [16]. Chest x-ray should be performed immediately after intubation to confirm the correct placement of the ETT.

Twelve percent of patients had newly diagnosed pneumonia after intubation. This could be due to the underlying respiratory failure or micro-aspiration after intubation. Visible aspiration was not reported on initial intubation in all patients.

### Immediate complication and mortality after intubation

Cardiac arrest was reported within 30 min of intubation in 2 patients. Both patients had an extensive cardiac and non-cardiac medical history. Additionally, both patients had exacerbation of their underlying disease requiring intubation. Patients were both induced with etomidate and rocuronium, were easily ventilated, and had an atraumatic intubation on first attempt without significant hypoxia that might have caused cardiac arrest. Most likely, the underlying disease was causing hemodynamic collapse and death.

Cardiac arrest during induction is reported to occur 0.7–11% of patients [5]. It is possible that cardiac arrest is a result of difficult intubation, leading to multiple attempts, resulting in hypoxia-driven bradycardia and possibly cardiac arrest. Additionally, Schwartz et al. reported a 3% mortality within 30 min of intubation [15] not necessarily related to the intubation itself. Most of the time the progression of underling disease was the major factor in mortality.

### In-hospital mortality and comorbidity on admission

The 30-day in-hospital mortality was 31.6% and the overall in-hospital mortality rate was 37.1% in our study population. The mortality rate reflects the overall very sick patient population and is most likely not associated with our intubation. There is no data in the literature about 30-day mortality or hospital stay of this specific patient population and we believe that this new data is important for hospital management and quality improvement.

In general, according to multicentre studies, the ICU mortality ranges from 8 to 17% [17–19]. Additionally, patients who are admitted to ICUs and survive hospitalization have a 1.3-times higher (14.1% vs. 10.9%) mortality rate in the six months after discharge. ICU survivors receiving mechanical ventilation had substantially increased 3-year mortality (57.6%) compared to non-ventilated patients (32.8%). Similarly, for those receiving mechanical ventilation, the risk was concentrated in the first 6 months after hospital discharge (6-month mortality, 30.1%). Additionally, patients who received mechanical ventilation during their hospitalization were more likely to have greater comorbidities compared with those who did not receive mechanical ventilation [20]. We believe that the mortality seen in our study is higher than the ICU mortality because the patients who required emergent intubation were overall more decompensated and had multiple comorbidities on admission. Further analysis comparing the comorbidity of the general admitted population to the comorbidity of the in-hospital intubated population might be helpful to identify the severity of disease and enable comparison with other data.

### Hospital and ICU stays

In our study, the mean hospital stay was 22 ± 20 days, and the mean ICU-stay was 14 days (13.9 ± 0.9, CI 12.1–15.8) with a 7.3% ICU-readmission rate which is significantly higher than the average ICU-stay reported in other studies. By comparison, Rosenberg et al. reported a mean ICU-stay of 4.6 days and hospital stay of 11.8 days [21]. Finkielman reported the median ICU-stay of 6.5 days [18] and Knaus et al. 3.3 to 7.3 days in a multicentre analysis including 42 ICUs [22]. Our study finding indicates that patients requiring emergent intubation have significantly longer ICU and hospital stays compared to the general ICU population. The aggregation of several diseases, complications, and operations could have accounted for the prolonged ICU-stay, in addition to prolonged mechanical ventilation. Factors that have been reported to influence ICU-stay include specific medical conditions, like sepsis or acute respiratory distress syndrome, the hospital discharge policy, and ICU staffing. ICU accounts for approximately 7% of total U.S. hospital beds and 20 to 30% of the hospital costs. Although differences in the intensity of treatment may lead to discrepancies, ICU-stay may be used as a surrogate measure of cost [23]. Identifying risk factors to decrease ICU-stay might help saving cost in the future.

### Airway management devices and technique

A supraglottic airway device was used in only 1 patient as a bridge to intubation. Supraglottic airway devices have been shown to be effective for airway rescues in emergent airway management. Sorbello M et al. reviewed different types supraglottic airway device use in different situations [24]. A bougie was used in 2 patients. Driver et el. described the use of bougie compared with an endotracheal tube and stylet resulted in significantly higher first-attempt intubation success among patients undergoing emergency endotracheal intubation [25]. The use of video-laryngoscopes for emergent airway management is associated with a lower number of intubation attempts and with a lower frequency of esophageal intubation [26] and thus, may reasonably be regarded as the first choice in emergent airway management. Like other airway management techniques, the use of rapid sequence intubation or cricoid pressure requires preparatory instruction and periodic training. The current literature is controversial and ss per Salem et al. investigations are warranted to determine the characteristics of the CP technique that maximize its effectiveness while avoiding the risk of airway-related complications in the various patient populations [27]. Ultimately the anesthesiologist needs to judge which device is most suitable by identifying the cause of difficult intubation in each patient. Additionally, anesthesiologist should use the airway technique that they are most experienced with and that is best for the individual situation. As with any intubation, practice and routine use will improve performance.

### Airway education

Airway education plays a crucial role preparing for emergent intubations in the hospital setting. Crisis management training, communication, leadership, team coordination, and shared understanding of roles has been shown to improve the success of airway management in emergency settings. We believe that the low complication rate of immediate airway-related complications, such as esophageal intubation, aspiration, and dental trauma, is most likely due to the extensive airway education and training at our institution. Early exposure to real situations combined with simulation and discussion sessions to review every possible scenario in non-operating room emergent airway management will train first responders to use appropriate clinical judgement. Additionally, upon response to an emergent airway management advanced planning, proper positioning, patient preparation, coupled with a strategy for both the intubation procedure and its rescue, are essential to minimize the complication rate.

Beyond that, the nontechnical aspect is important as well. The Difficult Airway Society (DAS) 2015 guidelines clearly introduce the concept of ‘stop-and-think’ magic words in their algorithm [28]. This concept is to be perceived as a handbrake encouraging us to slow down to automatic (intuitive) thinking in favor of the rational one, aimed at avoiding cognitive biases and to ignite the thinking out-of-the-box process [24].

### Limitations

It is difficult to generalize these findings since the approach to the airway management outside the OR is highly dependent on the hospital or institutional settings. Depending on institution, it could be an attending anaesthesiologist, a resident or a CRNA responding to an airway.

Although abundant information was collected on these patients, the retrospective nature of the analysis reveals some interesting relationships however causality of independent variables and risk factors cannot be inferred. The mortality analysis in this study was purely descriptive without analysis of causality or association to intubation we performed. Additionally, mortality is a poor measurement for causality because of the complexity of diseases in addition to many unidentifiable confounders.

Data collection from the intubation notes was a limiting factor. Only information that was pre-created as a check-off box was collected and analysed. There is a risk of underreporting of complications: the quality of the laryngoscopic view obtained, and the actual number of laryngoscopic attempts performed. Additionally, demographics like BMI, ASA status, comorbidity was recorded only on initial admission. There is potential that those demographics might have changed over the hospital course. Whether the demographic change is associated with worsening outcome should be evaluated in future studies.

## Conclusion

Emergent airway management outside of the OR is performed in a high-risk patient population with multiple comorbidities with high ASA scores on admission. Only a small number of intubation-related complications were reported. Most of the complications were related to the deconditioning of the patient’s physiologic state rather than the intubation procedure itself. Overall, with adequate training and education in the fundamentals of airway management, emergent airway management can be performed safely outside of the OR. Further studies are needed to identify individual predictors of reintubation rate, adverse outcome, and mortality for quality improvement.

## Data Availability

The datasets generated and/or analysed during the current study are not publicly available due to institutaional HIPPA (Health Insurance Portability and Accountability Act) policy, but are available from the corresponding author on reasonable request.

## Abbreviations

ASA: American Society of Anesthesiologist
BMI: Body Mass Index
CRNA: Certified Registered Nurse Anesthetist
EF: Ejection Fraction
ETT: Endotracheal Tube
ICD: Implantable Cardioverter Defibrillator
ICU: Intensive Care Unit
NPO: Nil Per Os
OR: Operating Room

## References

[1] Asai T (2018). Airway management inside and outside operating rooms-circumstances are quite different. Br J Anaesth.

[2] Divatia JV, Khan PU, Myatra SN (2011). Tracheal intubation in the ICU: life saving or life threatening?. Indian J Anaesth.

[3] Benedetto WJ, Hess DR, Gettings E (2007). Urgent tracheal intubation in general hospital units: an observational study. J Clin Anesth.

[4] Jaber S, Amraoui J, Lefrant JY (2006). Clinical practice and risk factors for immediate complications of endotracheal intubation in the intensive care unit: a prospective, multiple-center study. Crit Care Med.

[5] Mort TC (2004). Emergency tracheal intubation: complications associated with repeated laryngoscopic attempts. Anesth Analg.

[6] Shiga T, Wajima Z, Inoue T, Sakamoto A (2005). Predicting difficult intubation in apparently normal patients: a meta-analysis of bedside screening test performance. Anesthesiology..

[7] Stauffer JL, Olson DE, Petty TL (1981). Complications and consequences of endotracheal intubation and tracheostomy. Am J Med.

[8] Zwillich CW, Pierson DJ, Creagh CE, Sutton FD, Schatz E, Petty TL (1974). Complications of assisted ventilation. Am J Med.

[9] Martin LD, Mhyre JM, Shanks AM, Tremper KK, Kheterpal S (2011). 3,423 emergency tracheal intubations at a university hospital: airway outcomes and complications. Anesthesiology.

[10] Rochlen LR, Housey M, Gannon I, Mitchell S, Rooney DM, Tait AR, Engoren M (2017). Assessing anesthesiology residents' out-of-the-operating-room (OOOR) emergent airway management. BMC Anesthesiol.

[11] Cook TM, Woodall N, Frerk C (2011). Fourth National Audit Project: major complications of airway management in the UK: results of the fourth National Audit Project of the Royal College of Anaesthetists and the difficult airway society. Part 1: Anaesthesia. Br J Anaesth.

[12] Tachibana N, Niiyama Y, Yamakage M (2015). Incidence of cannot intubate-cannot ventilate (CICV): results of a 3-year retrospective multicenter clinical study in a network of university hospitals. J Anesth.

[13] McCoy EP, Russell WJ, Webb RK (1997). Accidental bronchial intubation. An analysis of AIMS incident reports from 1988 to 1994 inclusive. Anaesthesia..

[14] Dronen S, Chadwick O, Nowak R (1982). Endotracheal tip position in the arrested patient. Ann Emerg Med.

[15] Schwartz DE, Matthay MA, Cohen NH (1995). Death and other complications of emergency airway management in critically ill adults. A prospective investigation of 297 tracheal intubations. Anesthesiology..

[16] Pollard RJ, Lobato EB (1995). Endotracheal tube location verified reliably by cuff palpation. Anesth Analg.

[17] Zimmerman JE, Kramer AA, Knaus WA (2013). Changes in hospital mortality for United States intensive care unit admissions from 1988 to 2012. Crit Care.

[18] Finkielman JD (2004). Morales lJ. Peters SG et al Mortality rate and length of stay of patients admitted to the intensive care unit in July Crit Care Med.

[19] Kuijsten HA, Brinkman S, Meynaar IA (2010). Hospital mortality is associated with ICU admission time. Intensive Care Med.

[20] Wunsch H, Guerra C, Barnato AE, Angus DC, Li G, Linde-Zwirble WT (2010). Three-year outcomes for Medicare beneficiaries who survive intensive care. JAMA.

[21] Rosenberg AL, Zimmerman JE, Alzola C, Draper EA, Kmaus WA (2000). Intensive care unit length of stay: recent changes and future challenges. Crit Care Med.

[22] Knaus WA, Wagner DP, Zimmerman JE, Draper EA (1993). Variations in mortality and length of stay in intensive care units. Ann Intern Med.

[23] Rapoport J, Teres D, Zhao Y, Lemeshow S (2003). Length of stay data as a guide to hospital economic performance for ICU patients. Med Care.

[24] Sorbello M, Petrini F (2017). Supraglottic airway devices: the search for the best insertion technique or the time to change our point of view?. Turk J Anaesthesiol Reanim.

[25] Driver BE, Prekker ME, Klein LR (2018). Effect of use of a Bougie vs endotracheal tube and Stylet on first-attempt intubation success among patients with difficult airways undergoing emergency intubation: a randomized clinical trial. JAMA.

[26] Rombey T, Schieren M, Pieper D (2018). Video versus direct laryngoscopy for inpatient emergency intubation in adults. A Systematic Review and Meta-Analysis of Randomized Controlled Trials Dtsch Arztebl Int.

[27] Salem MR, Khorasani A, Zeidan A, Crystal GJ (2017). Cricoid pressure controversies: narrative review. Anesthesiology..

[28] Sorbello M, Afshari A, De Hert S (2018). Device or target? A paradigm shift in airway management with implications for guidelines, clinical practice and teaching. Eur J Anaesthesiol.

